# From Paper to Pixels—A Narrative Review of Occlusal Analysis

**DOI:** 10.3390/dj14090573

**Published:** 2026-09-07

**Authors:** May Aljanahi, Gerry Mckenna, Keyvan Moharamzadeh, Haitham Elbishari

**Affiliations:** 1 Hamdan Bin Mohammed College of Dental Medicine (HBMCDM), Mohammed Bin Rashid University of Medicine and Health Sciences (MBRU), Dubai Health, Dubai P.O. Box 505055, United Arab Emirates; may.aljanahi@dubaihealth.ae (M.A.);; 2 School of Medicine, Dentistry and Biomedical Sciences, Queen’s University Belfast, Belfast BT9 7BL, UK; 3 Centre for Public Health, School of Medicine, Dentistry and Biomedical Sciences, Queen’s University Belfast, Belfast BT9 7BL, UK; 4 Faculty of Biology, Medicine and Health, School of Medical Science, Division of Dentistry, University of Manchester, Manchester M13 9PL, UK

**Keywords:** occlusal analysis, digital occlusal analysis, conventional methods, occlusal contact points, articulating paper, digital dentistry, bite force analysis

## Abstract

**Background/Objectives**: Occlusal analysis plays a key role in restorative and prosthodontic treatment, influencing diagnosis, treatment planning, and long-term outcomes. This narrative review provides an overview of conventional and digital methods for occlusal analysis and their clinical applications. **Methods**: A literature search was conducted using PubMed/MEDLINE, Scopus, and Google Scholar to identify relevant publications on conventional and digital occlusal analysis. The retrieved literature was reviewed and synthesized narratively. **Results**: Conventional methods, including articulating paper, shimstock foil, films, sprays, and waxes, remain widely used because of their simplicity and accessibility but are limited by subjective interpretation and the inability to quantify occlusal forces. Digital technologies, including T-Scan, OccluSense, Accura, intraoral scanners, virtual articulators, and jaw-tracking systems, provide objective information on occlusal contacts, force distribution, timing, and mandibular movements. However, their use may be limited by cost, technical requirements, and operator training. **Conclusions**: Conventional methods remain valuable in daily clinical practice, while digital technologies provide objective data that can enhance occlusal assessment and treatment planning. As digital workflows continue to evolve, structured training and standardized clinical protocols will be important for their successful integration into clinical practice.

## 1. Introduction

Mastication is a vital function of the oral system, dependent on harmonious occlusion and balanced force distribution. The role of occlusion in maintaining oral health has long been recognized, underpinning diagnosis, treatment planning, and the long-term success of prosthetic and restorative interventions [1]. Dental occlusion is defined as “the static relationship between the incising or masticating surfaces of the maxillary or mandibular teeth or tooth analogs”, whereas dynamic occlusion refers to the contacts that occur during mandibular movements, playing a critical role in mastication, speech, and overall oral function [2].

Accurate occlusal assessment has therefore become a cornerstone of modern dental care, particularly in implant-supported prostheses, where complications such as ceramic fracture and peri-implant bone loss are strongly associated with occlusal overload [3,4,5,6]. Disruption of static or dynamic relationships can also lead to chewing difficulties, temporomandibular disorders, and biomechanical complications, including restoration fracture and periodontal strain [7,8].

The evaluation of mandibular movements has been investigated since the 19th century, with Posselt’s envelope of motion remaining a key reference in prosthodontics [9,10,11,12]. Conventional methods such as articulating paper, foils, sprays, and waxes continue to be widely used due to simplicity and affordability [13,14,15,16]. However, they provide only qualitative information and cannot capture the timing or magnitude of occlusal forces.

Parallel to these challenges, dentistry has undergone a digital transformation. Intraoral scanners, CAD/CAM, 3D printing, and guided implant surgery have improved precision, efficiency, and patient experience [17,18]. Within this context, occlusal analysis has also advanced. Digital systems such as T-Scan and Modjaw provide objective, quantifiable data on occlusal force, timing, and distribution, improving reproducibility and enabling refined prosthetic design [19,20,21,22,23].

Although digital systems offer objective, quantifiable data, conventional methods remain dominant because of their accessibility and the limited clinical validation of digital techniques. This narrative review therefore examines both conventional and digital approaches to occlusal analysis, outlining their underlying principles, advantages, limitations, and clinical relevance in restorative and prosthodontic practice.

## 2. Literature Search Strategy

A literature search was conducted using PubMed/MEDLINE, Scopus, and Google Scholar to identify publications relevant to conventional and digital occlusal analysis. The search was performed between 5 January 2025 and 12 January 2026 and was updated throughout manuscript preparation to include studies published up to 12 January 2026. Search terms included “occlusal analysis”, “digital occlusal analysis”, “occlusal contacts”, “articulating paper”, “shimstock”, “occlusal indicators”, “T-Scan”, “OccluSense”, “Accura”, “Modjaw”, “jaw tracking”, “virtual articulator”, and “intraoral scanner”.

Peer-reviewed clinical studies, laboratory investigations, systematic reviews, and narrative reviews relevant to conventional and digital occlusal analysis were considered for inclusion. Publications not directly relevant to the review topic and duplicate records were excluded. The retrieved literature was reviewed for relevance to the objectives of this review, with particular consideration given to clinical studies, review articles, and recent advances in conventional and digital occlusal assessment. Additional publications were identified through manual screening of the reference lists of relevant articles. As this was a narrative review, the search was intended to provide a broad overview of the available literature and current developments in the field rather than a systematic synthesis of the evidence.

## 3. Conventional Methods for Occlusal Analysis

Occlusal contacts have traditionally been evaluated using conventional clinical indicators such as articulating paper, shimstock foil, articulating films, waxes, sprays, and silk strips [24,25,26,27]. These methods remain central to everyday dental practice because of their accessibility, ease of application, and long-standing clinical use. However, they are inherently limited by subjectivity and cannot provide quantitative data regarding occlusal force or contact timing [15,28].

### 3.1. Articulating Paper

Articulating paper is the most widely used method in dentistry and has been employed for over a century [15,28,29,30]. It is available in different thicknesses, shapes, and colors, designed to record static contacts and interferences during mandibular closure. The markings are straightforward to visualize and require minimal chairside time, which explains its popularity. However, interpretation remains subjective, as the size or intensity of the marks does not reliably correspond to occlusal force [29,30].

The thickness of articulating paper significantly influences accuracy and reliability. Papers range from 8 µm to 200 µm, and the choice of thickness can alter clinical outcomes [27]. When adjusting occlusion, thinner papers (8–12 µm) are often preferred because they detect fine, high-pressure contacts without altering the occlusal relationship. These thin varieties are particularly valuable in prosthodontic treatment, where precision is critical. In contrast, thicker papers (80–200 µm) produce wider and more visible marks but risk exaggerating contact areas and may result in unnecessary adjustment [31,32,33]. Understanding the characteristics of each thickness is therefore essential to avoid misinterpretation.

A common clinical belief is that broader or darker marks indicate heavier forces, while smaller or lighter marks reflect minimal contacts. However, research has consistently shown that this assumption is unreliable, with studies demonstrating little or no correlation between articulating paper mark size and true occlusal load [31,32]. Other studies have further confirmed that mark size and intensity are not consistently correlated with true occlusal load [15,34,35,36].

**Figure 1:**
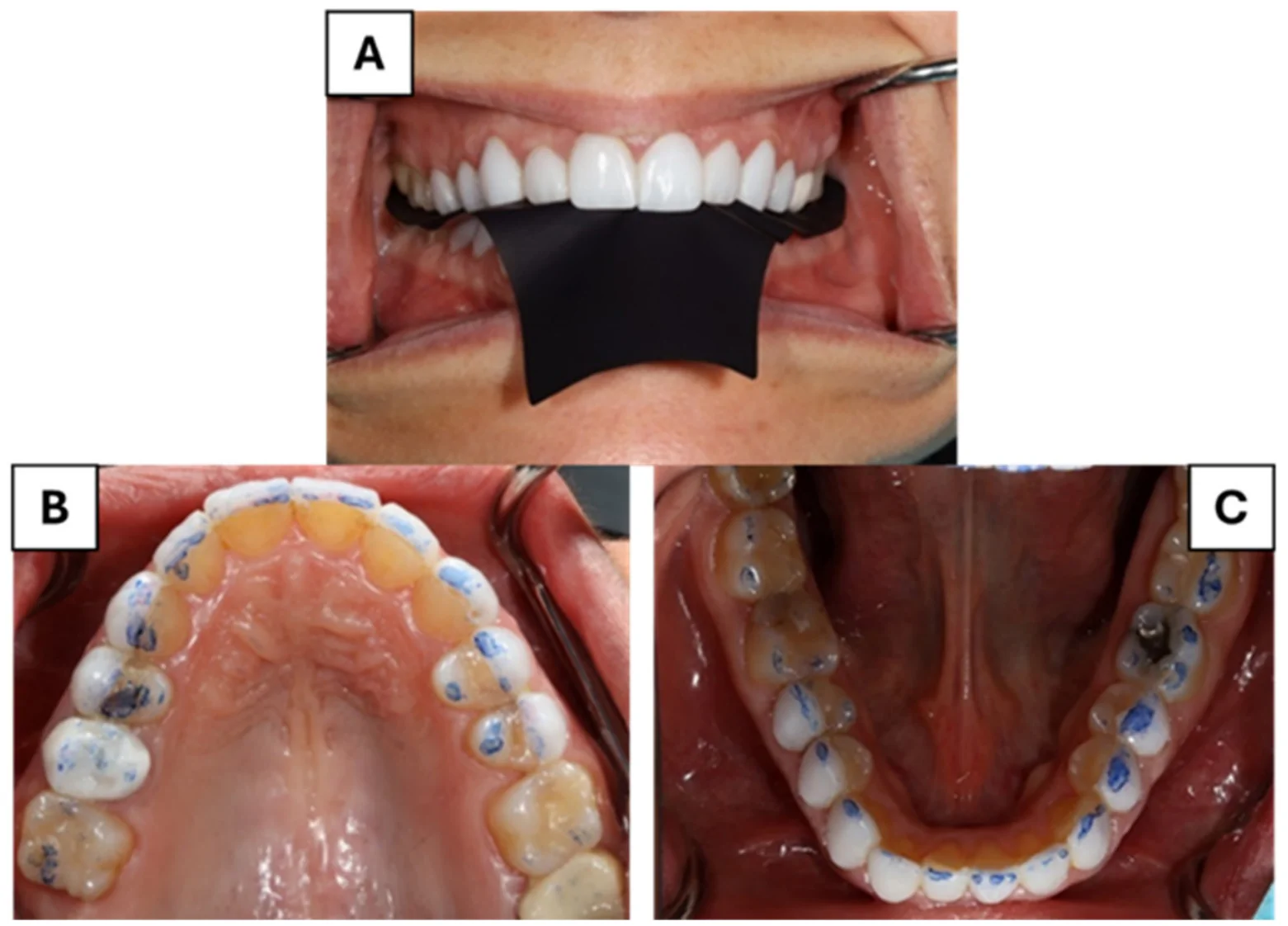
(**A**) U-shaped articulating paper to assess occlusion. (**B**) Occlusal contacts were visualized on the veneer during assessment of static occlusion. (**C**) Heavy occlusal contacts were observed on the lower left side.

Although its diagnostic accuracy is limited, articulating paper remains a widely adopted and practical tool for preliminary occlusal assessment in daily practice. Figure 1A shows articulating paper applied intraorally, while Figure 1B,C demonstrate the markings on a veneer during static occlusal assessment, where heavy contacts were identified on the lower left quadrant.

### 3.2. Shimstock

Shimstock foil, typically 8 µm thick, is another common method for evaluating occlusion [34,37]. Unlike articulating paper, it does not leave visible markings but instead relies on tactile assessment. The foil is placed between occluding teeth during mandibular closure, and resistance to withdrawal indicates the presence of contact. If the foil passes freely, no contact is assumed.

This method is particularly useful for confirming occlusal contacts and for verifying occlusion following restorative procedures. Due to its minimal thickness, it can detect very light contacts that might be missed by thicker materials such as articulating paper [38]. However, shimstock foil cannot identify the location, magnitude, or distribution of occlusal forces, and outcomes remain dependent on clinician judgment. As such, its use is best suited for specific cases rather than comprehensive occlusal analysis.

**Figure 2:**
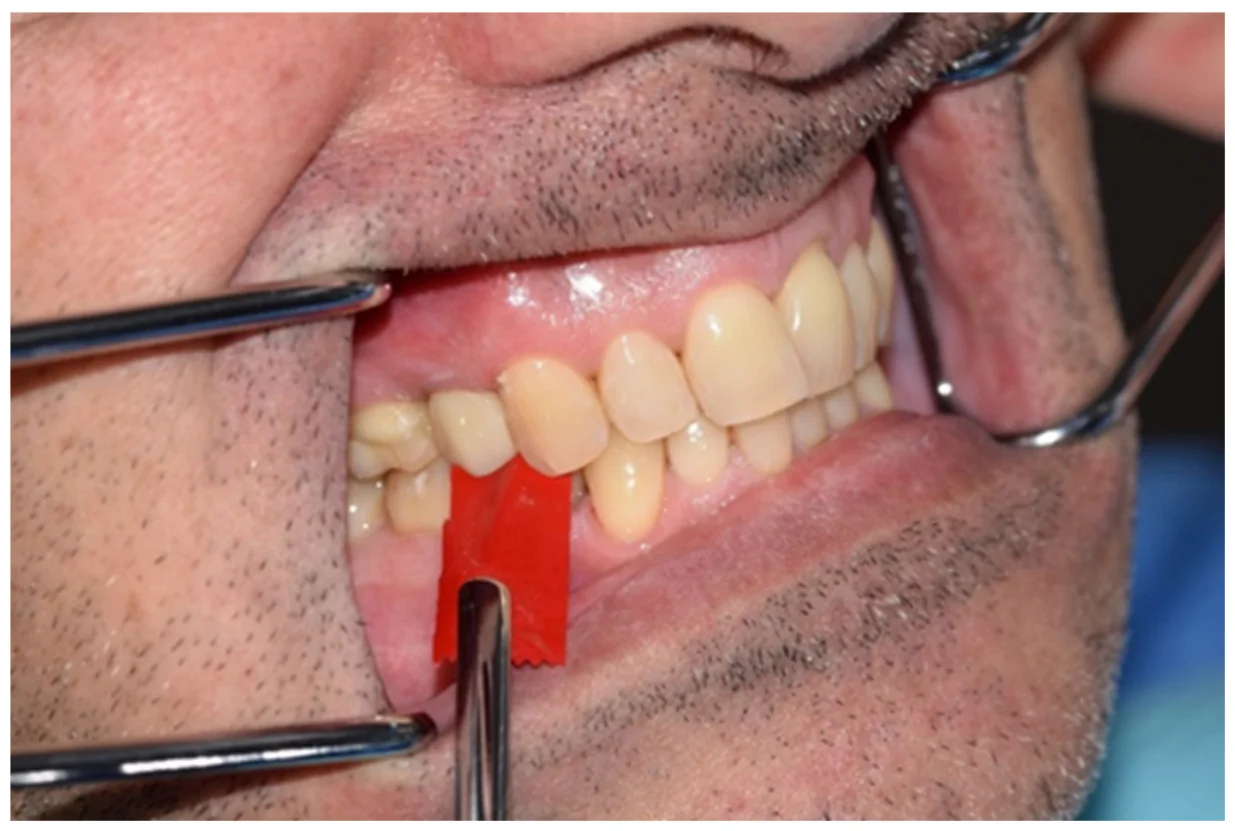
Clinical application of shimstock foil to assess occlusal contact in the maxillary premolar region during mandibular closure.

While shimstock provides valuable confirmation of occlusal contact, it should be regarded as a complementary method rather than a comprehensive diagnostic tool. Figure 2 illustrates the use of shimstock foil in the maxillary premolar region during mandibular closure.

### 3.3. Articulating Films and Foils

Articulating films and foils were developed to achieve greater accuracy than conventional paper, particularly in prosthodontic procedures involving smooth or highly polished surfaces such as metal, ceramic, or implant-supported restorations, where traditional indicators may be less effective [37].

Both materials are extremely thin, often around 8 µm, which enables highly accurate detection of even minimal occlusal contacts [25,39]. Their slim profile reduces the risk of altering the patient’s bite or creating false-positive marks, problems more common with thicker indicators. As a result, they are especially advantageous during the try-in and final adjustment stages of restorations, where precision is essential.

Although articulating films and foils are often used interchangeably in clinical practice due to their similar function and thickness, films are typically polyester-based materials coated with pigment, whereas foils are thin metal-based sheets. Both are designed to provide highly accurate occlusal markings, particularly on polished or moisture-prone surfaces.

These materials generally consist of double-sided, color-coated sheets that can mark even in the presence of saliva. Some products, such as Arti-Fol (Bausch Inc., Cologne, Germany), incorporate wetting agents that enhance adhesion to moist surfaces, ensuring clearer and more reliable transfer of markings. Both films and foils are available in a variety of colors and can be used intraorally or on diagnostic models in laboratory settings.

Despite these advantages, films and foils often require special holders for accurate intraoral placement. On excessively wet or glossy surfaces, markings may appear faint unless sufficient occlusal pressure is applied.

These materials are therefore most valuable in the fine adjustment of polished prostheses or implant-supported restorations, where high precision and reliability are required.

### 3.4. Occlusal Sprays

Occlusal sprays, such as Arti-Spray (Bausch GmbH, Cologne, Germany) and Occlude (Pascal International, Bellevue, WA, USA), are applied as a thin, colored coating on the occlusal surfaces of teeth. When the patient bites down, the sprayed layer is rubbed off at points of contact, leaving visible markings that can be easily identified. These sprays are particularly useful for verifying the fit of crowns and bridges and for detecting occlusal interferences during the trial stage and before cementation [16].

They are also valuable in highlighting heavy interproximal contact between adjacent crowns or between a crown and natural tooth structure, where other indicators may be less effective.

One limitation is that saliva or an excessive application of spray can obscure results, producing unclear markings or false positives. Since they only indicate the presence of contact but cannot provide information on force magnitude or contact timing, occlusal sprays are generally best used in combination with other methods.

Occlusal sprays therefore serve as a convenient adjunct for evaluating crown and bridge fit and interproximal contacts, but they should be supplemented with additional indicators for a comprehensive occlusal assessment.

### 3.5. Articulating Silk Strips

Articulating silk strips are composed of natural silk fibers and are designed to provide flexibility, durability, and resistance to tearing. Compared with articulating paper, silk adapts more effectively to the complex cusp-fossa morphology of teeth, reducing the likelihood of pseudo-marks and enhancing the accuracy of occlusal contact registration [25].

A major advantage of silk strips is their high dye capacity, which enables multiple contacts to be recorded without frequent replacement. At approximately 80 µm in thickness, they are also comfortable for patients and are particularly effective in registering contacts in narrow occlusal areas where other materials may be less adaptable [16].

Clinically, they are especially useful during adjustments of intricate prosthodontic restorations, such as full-contour crowns and implant-supported prostheses, where both adaptability and durability are required. However, silk strips lose effectiveness once the dye dries, and as with all visual indicators, their interpretation remains subjective.

Articulating silk strips are therefore best considered as a durable and adaptable adjunct for occlusal marking in complex restorative cases, but they should not be relied upon as a standalone diagnostic method. A summary of conventional occlusal analysis methods, including their characteristics, applications, features, and limitations, is presented in Table 1.

## 4. Digital Occlusal Analysis

Digital occlusal analysis began in 1984 with the introduction of the T-Scan system (Tekscan, Norwood, MA, USA), which represented a milestone in occlusal diagnostics [40]. Unlike traditional static marking methods, T-Scan provided a computerized approach that enabled clinicians to assess occlusal contact timing and force distribution with greater precision [16]. For the first time, premature contacts could be objectively identified, offering deeper insights into functional occlusion. Since its introduction, multiple studies have evaluated digital occlusal analysis systems, consistently highlighting their reliability and reproducibility in clinical settings [41,42,43,44].

In 2004, T-Scan evolved into T-Scan III, offering improved resolution and advanced software capable of presenting occlusal forces in three-dimensional form. This development significantly enhanced its clinical value, particularly in implant dentistry and complex prosthodontic case planning [45]. In 2015, T-Scan Novus was introduced, featuring refined ergonomics and integration with digital impression workflows. Owing to continuous upgrades and a substantial body of research support, T-Scan remains the most extensively validated and widely studied system for digital occlusal assessment, and it continues to serve as the benchmark for computerized occlusal analysis [45].

Several alternative systems were later developed, often with the aim of simplifying the procedure or offering more cost-effective solutions. Accura, launched in South Korea in 2017 (Dmetec, Bucheon, Republic of Korea), generates two-dimensional visual maps of occlusal forces, though it does not record timing. Despite this limitation, it has demonstrated accuracy comparable to T-Scan in assessing contacts in maximum intercuspation [46], making it a more accessible option for routine use even if its lack of dynamic analysis restricts its application in complex restorative or implant cases.

OccluSense, introduced in Germany in 2018 (Bausch GmbH, & Co. KG, Cologne, Germany), employs wireless, pressure-sensitive foils that transmit occlusal data to a tablet. These foils capture both static and dynamic contacts, presented in color-coded maps. Although the system does not quantify absolute force values, its portability and ease of use make it practical for everyday practice, positioning it as a convenient hybrid between conventional indicators and digital systems, particularly valuable in routine chairside applications where cost and simplicity are priorities [47].

Other systems have approached occlusion from different perspectives. In Italy, Teethan introduced surface electromyography (sEMG) to evaluate muscular activity during occlusion. By analyzing muscle activation patterns, the system provides indirect insight into functional balance, offering a useful adjunct for the diagnosis of muscle-related conditions such as temporomandibular disorders [48].

Earlier innovations also played a role in shaping digital occlusal analysis. In the 1990s, Dental Prescale (GC, Tokyo, Japan) became one of the first systems to use pressure-sensitive sheets to record bite force and contact area. Although limited to static occlusion, it has been widely used in research, particularly in implant load monitoring and bruxism studies [49]. The updated Dental Prescale II, launched in 2018, added calibration features, enabling bite force to be measured in Newtons and contact area in mm^2^ with greater accuracy [41]. While it remains primarily a research tool, its ability to quantify static force and contact area offers valuable information in specific clinical contexts.

Digital jaw-tracking technologies also emerged during this period, providing further insight into mandibular dynamics. The ARCUSdigma II system (KaVo, Biberach, Germany), launched in the mid-1990s, used ultrasonic sensors to record mandibular movement and condylar pathways. In 2012, this system was upgraded to ARCUSdigma III, which employed infrared technology to improve accuracy and allowed integration with virtual articulators in CAD/CAM workflows. Similarly, Zebris for Ceramill (Amann Girrbach, Pforzheim, Germany) employed optical tracking to simulate jaw function within CAD/CAM systems. Although both devices contribute valuable functional data, evidence regarding their clinical reproducibility remains limited, confining their role largely to academic and laboratory applications.

A more recent development, Modjaw (Modjaw SAS, Villeurbanne, France), launched in 2020, offers real-time “4D” tracking of mandibular movements. This technology records functional activities such as lateral excursions, protrusion, and chewing cycles, enabling clinicians to visualize occlusion dynamically during function. By integrating these records into digital workflows, Modjaw has proven especially useful in full-mouth rehabilitations, enhancing the accuracy of prosthetic design and demonstrating the growing importance of dynamic, patient-specific occlusal analysis in advanced prosthodontics [50].

In addition, intraoral scanners (IOS) have increasingly incorporated occlusal visualization features. Systems such as the CEREC Omnicam (Dentsply Sirona, Bensheim, Germany) and Medit i700 (Medit Corp., Seoul, Republic of Korea) can identify occlusal contacts in maximum intercuspation. While not designed specifically for occlusal analysis, their integration into digital restorative workflows makes them valuable for treatment planning and prosthesis fabrication. However, because IOS devices do not record occlusal force or timing, they should be regarded as complementary tools rather than replacements for dedicated occlusal analysis systems [44].

**Figure 3:**
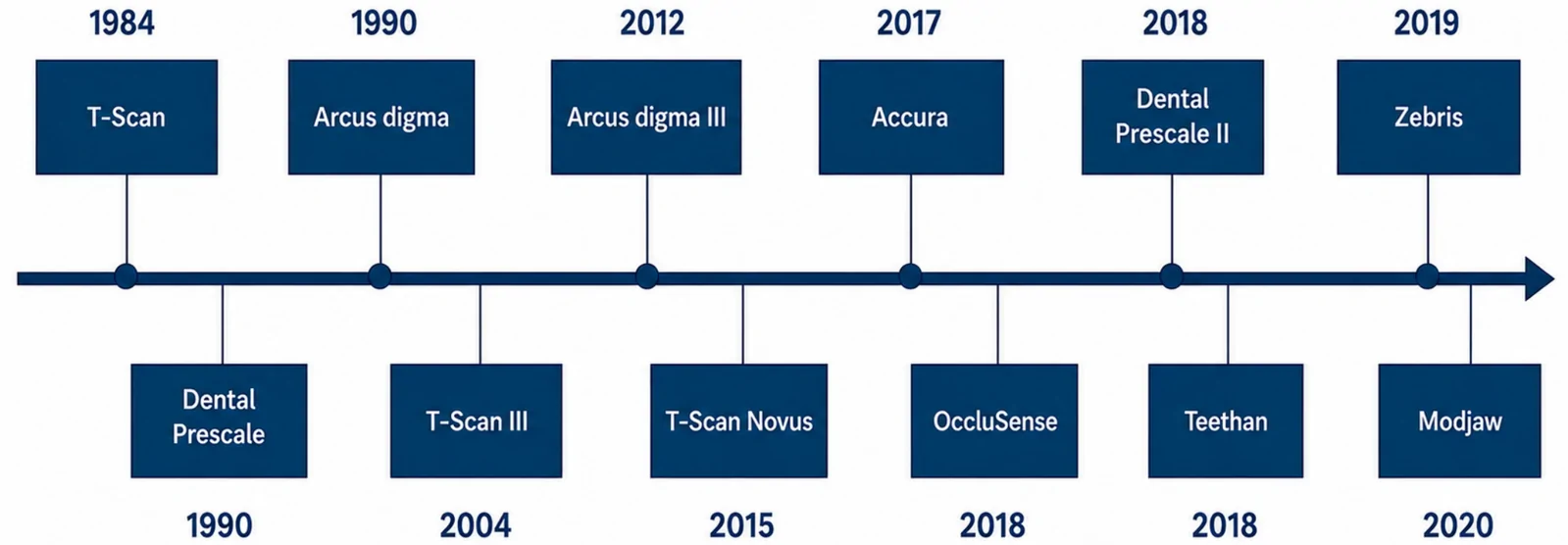
Illustrates the evolution towards digital occlusal analysis systems, presenting a timeline of key advancements over the years.

Taken together, the evolution of digital occlusal analysis illustrates a transition from static pressure-based methods to technologies capable of recording contact distribution, force, and mandibular kinematics in real time. This trajectory highlights dentistry’s broader shift toward precision, efficiency, and personalized care. Figure 3 presents a timeline of the development of these digital systems.

### 4.1. Contemporary Digital Systems for Occlusal Analysis and Jaw Tracking

The digitization of occlusal analysis has progressed markedly, transitioning from static marking techniques to advanced systems capable of real-time, dynamic assessment. Contemporary digital technologies provide quantifiable data, three-dimensional visualizations, and enhanced clinical integration, thereby offering advantages that may support their adoption over conventional methods. Some systems incorporate articulating paper with embedded sensors of varying thicknesses; for instance, T-Scan employs a 90 µm sensor, whereas the Dental Prescale System utilizes sheets up to 150 µm. These devices not only improve diagnostic accuracy but also facilitate complex prosthodontic, orthodontic, and implant treatment planning.

### 4.2. Computerized Digital Occlusal Analysis

**Figure 4:**
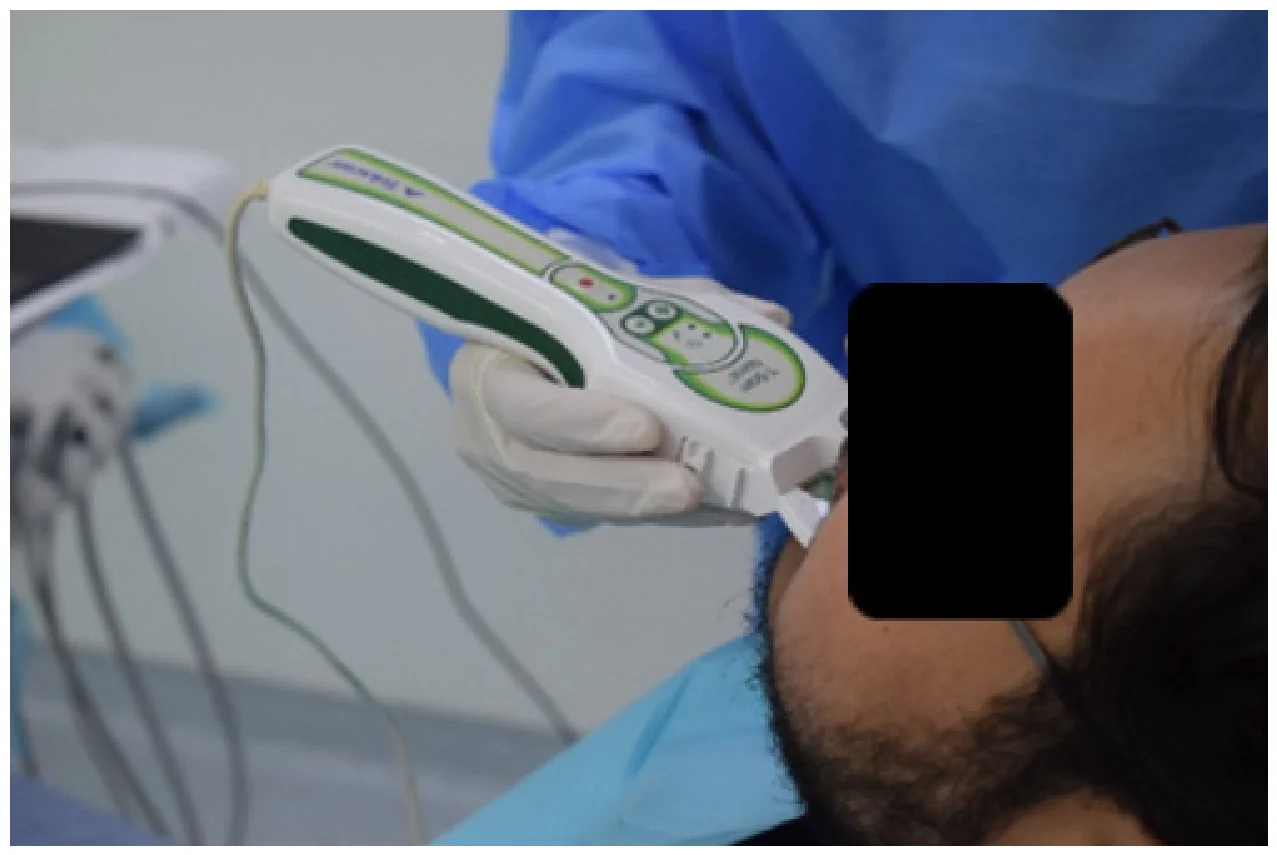
Digital occlusal analysis using the T-Scan system.

**Figure 5:**
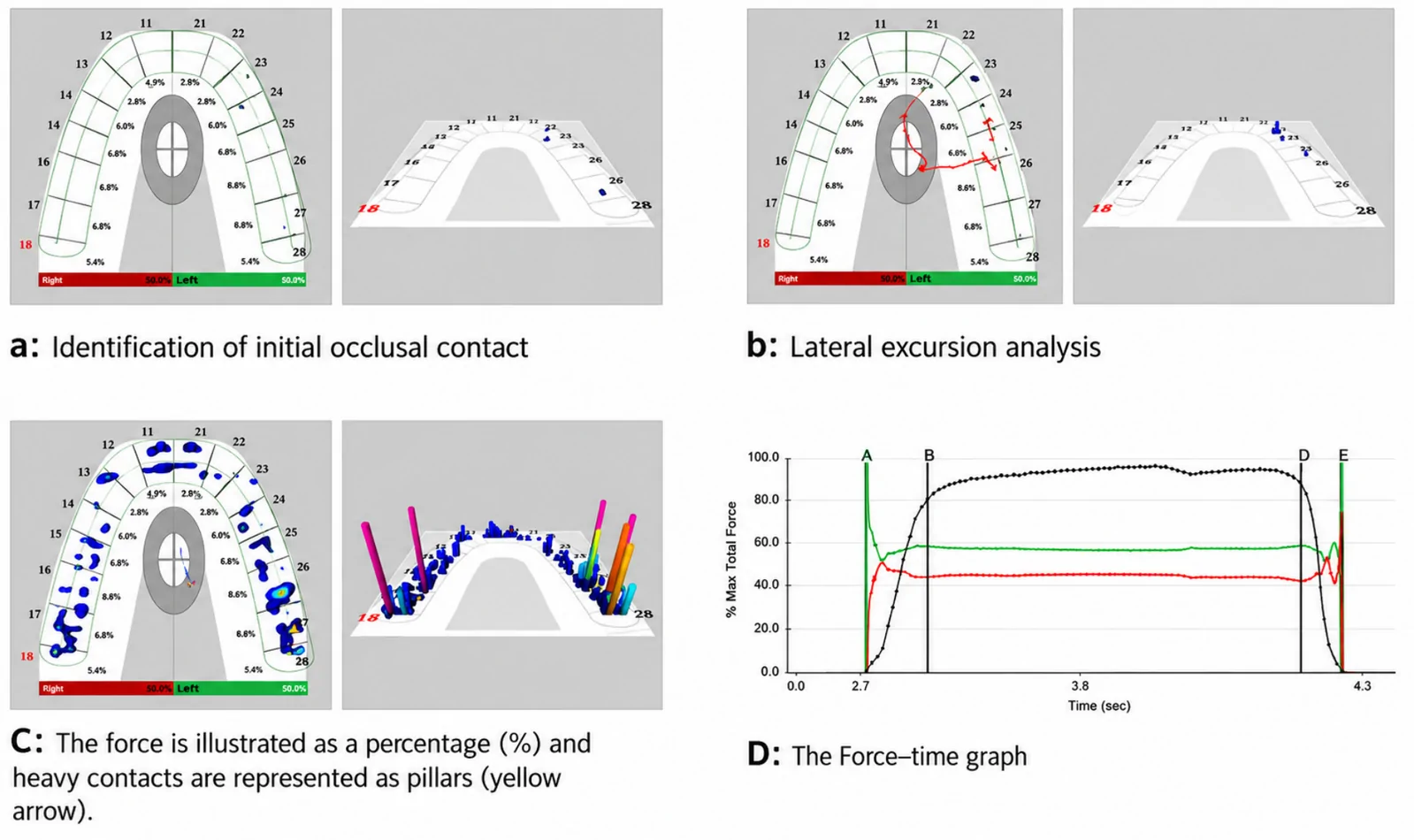
Interpretation of data analyzed using a computerized analysis system (T-Scan).

Computerized occlusal analysis enables detailed assessment of occlusal forces under both static and functional conditions. This section reviews currently available digital systems, their clinical applications, and diagnostic capabilities. Among these, T-Scan (Tekscan, USA) is the most extensively validated and widely studied device (Figure 4). Systems such as T-Scan, OccluSense, and Accura (Dmetec, Republic of Korea) employ thin intraoral sensors to digitally record occlusal contacts and force distribution. For example, the T-Scan sensor connects to a computer via USB and provides interactive two- and three-dimensional visualizations of occlusal dynamics [51]. Clinicians can assess occlusal harmony, identify premature contacts, and evaluate force distribution across the dentition, thereby enhancing both diagnostic precision and patient communication [52]. In addition, recordings can be exported in video format, allowing detailed analysis of force progression during functional movements (Figure 5).

T-Scan enables comprehensive evaluation of occlusal force dynamics and mandibular movements, with outputs generated as high-resolution images and video sequences for detailed post-analysis. This capability is illustrated in a clinical case (Figure 4), where digital occlusal analysis was conducted in a patient with temporomandibular disorder (TMD) who complained of chewing predominantly on one side. The recordings revealed early occlusal contact, a left lateral excursion pattern, and changes in force distribution over time. The accompanying force–time graph captured key occlusal events, including initial contact, maximum intercuspation, and subsequent disclusion. This illustrative case highlights the potential clinical utility of digital occlusal analysis for identifying functional occlusal discrepancies and informing individualized treatment planning.

T-Scan is the most extensively validated system in this category. One study demonstrated its high reproducibility, particularly in the assessment of implant occlusion and post-restorative adjustments [49]. Another investigation found that T-Scan significantly outperformed articulating paper in detecting force discrepancies and identifying the timing of initial contact during occlusal equilibration procedures [53].

Accura (Dmetec, Republic of Korea) is a more recent innovation that employs a U-shaped polyimide sensor to visualize static occlusal contact distribution. Although it does not record real-time timing or force sequences, it produces clear color-based maps reflecting contact intensity. A comparative study reported that Accura achieved accuracy comparable to T-Scan in identifying contacts at maximum intercuspation, with high repeatability across test conditions [46,54]. Its simplified interface and relative affordability may make it attractive for routine use in practices where detailed dynamic data are not required.

OccluSense (Bausch, Germany) integrates pressure-sensitive foil with wireless data transmission to a tablet interface. It displays occlusal contacts as color-coded pressure maps but does not measure absolute force values in Newtons or quantify occlusal timing. Consequently, its output is more qualitative than quantitative. While capable of recording both static and dynamic contact patterns, its resolution is lower than that of systems such as T-Scan. According to a recent study, results improved when the sensor was optimally positioned, yet variability remained a limiting factor [47]. In summary, digital occlusal force systems represent a significant advancement from traditional, subjective marking techniques toward data-driven clinical decision-making. T-Scan remains the benchmark in terms of diagnostic precision and research validation, particularly in prosthodontics and implant dentistry. In contrast, Accura and OccluSense provide more accessible alternatives, each offering unique advantages and limitations depending on the clinical context.

### 4.3. Virtual Articulators and Digital Facebows

Transferring the position of the maxillary arch in relation to craniofacial landmarks is a key step in occlusal assessment and prosthodontic treatment planning. Traditionally, this has been carried out using analog facebows to orient the upper jaw for mounting on mechanical articulators. These devices rely on three anatomical reference points—two posterior and one anterior—to help reproduce the arc of mandibular closure and provide an approximate indication of the occlusal plane [55].

**Figure 6:**
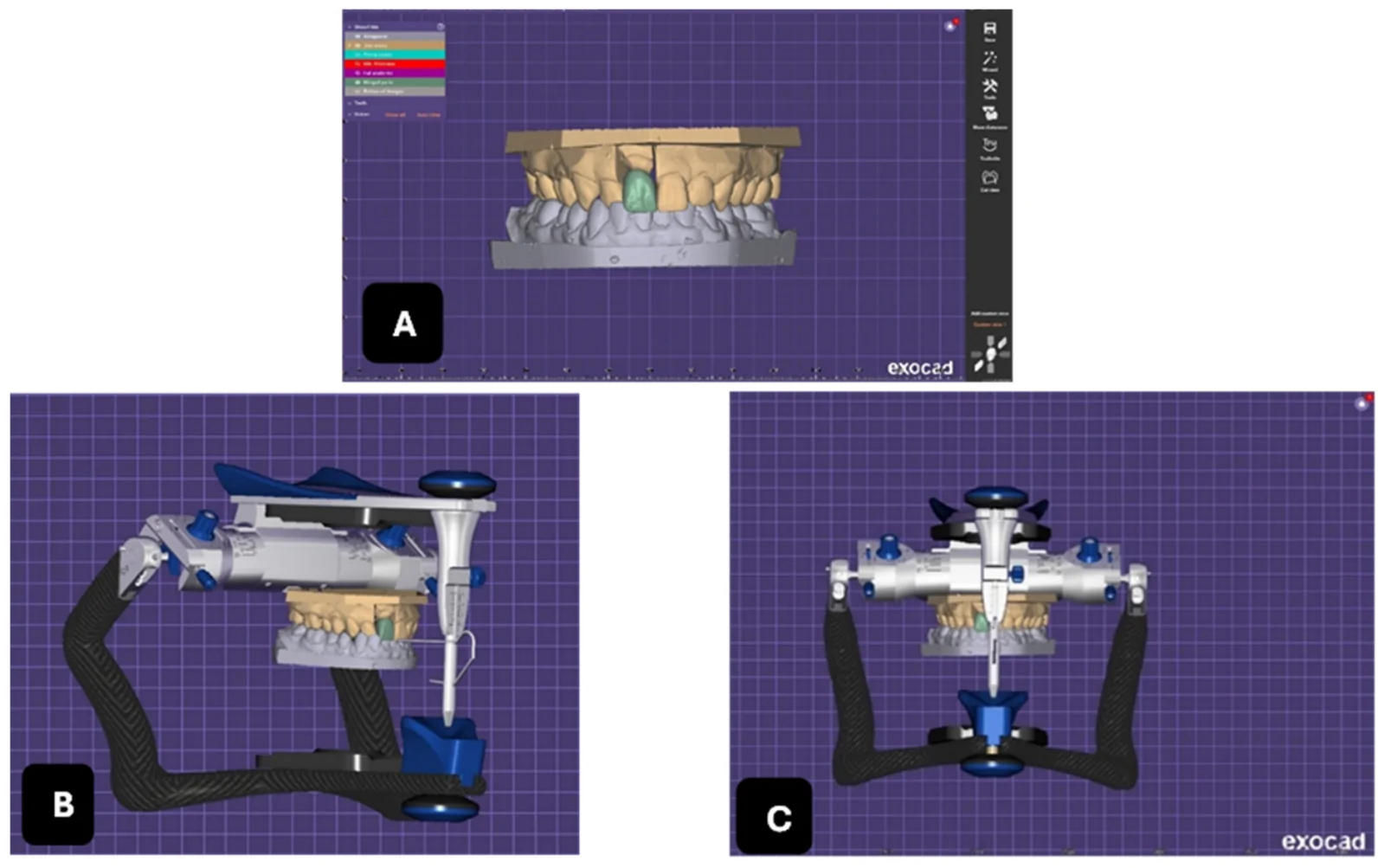
Digital prosthodontic planning using exocad of casts in intercuspal position (ICP). (**A**) Crown design for the upper right central incisor with occlusion evaluated in the digital environment. (**B**) Digital casts mounted using a virtual facebow are shown in a lateral view within the articulator simulation. (**C**) Frontal view of the digitally mounted casts, illustrating occlusal alignment and analysis.

In digital dentistry, this process has evolved through the incorporation of advanced imaging and motion capture technologies, such as CBCT, facial scanning, intraoral scanners, jaw-tracking systems, and digital photography [55,56]. These tools now allow clinicians to carry out the transfer virtually, within CAD software platforms like exocad and 3Shape [57] (Figure 6). Some systems use reference planes, such as the Frankfort horizontal or axis-orbital line, to help guide the spatial positioning of the virtual maxillary cast [23,58,59].

Accurate occlusal analysis begins with correctly transferring the spatial position of the maxillary arch relative to craniofacial landmarks. Whether using analog or digital articulators, this transfer is fundamental to ensure reliable evaluation of occlusal contacts, force distribution, and mandibular movement within both static and functional simulations. As digital workflows become more common, the transition to virtual articulation plays a critical role in modern occlusal analysis.

Virtual mounting can be achieved using several techniques, including digital facebows, dentofacial analyzers, or simple spatial centering within the software environment [18,60,61,62]. These methods rely on digital data to replicate anatomical orientation. Digital photographs, lateral cephalometric radiographs, CBCT, and three-dimensional (3D) facial scans are commonly used to record spatial references [61,62].

Although digital photography is widely used to capture horizontal orientation, its 2D nature limits its ability to reflect full spatial relationships [63]. Facial scanners, on the other hand, enable a more detailed three-dimensional recording. The process typically involves two main steps: registering the reference plane and aligning the facial scan with the intraoral model [18,58]. The Frankfort horizontal, axis-orbital, or true horizontal planes are often used during this alignment, although the scanner’s resolution and the merging protocol directly influence the outcome [64].

CBCT imaging is another method that allows for virtual cast transfer. Like facial scanning, the process involves recording a reference plane and then digitally aligning the maxillary arch within the CBCT dataset [64,65]. The Frankfort horizontal and axis-orbital planes are most commonly used for this technique. One study comparing CBCT-based virtual transfer with traditional facebow mounting found that the virtual method produced more consistent positioning, with less variability in cast placement [58]. Additionally, casts in the virtual group were slightly more anteriorly positioned, and the occlusal plane angle was less steep than in the analog group, suggesting a more predictable result.

While traditional articulators replicate static positions, jaw-tracking technologies now allow occlusal relationships to be evaluated under dynamic conditions, further advancing the precision of digital occlusal analysis.

### 4.4. Intraoral Scanners and Occlusal Analysis

**Figure 7:**
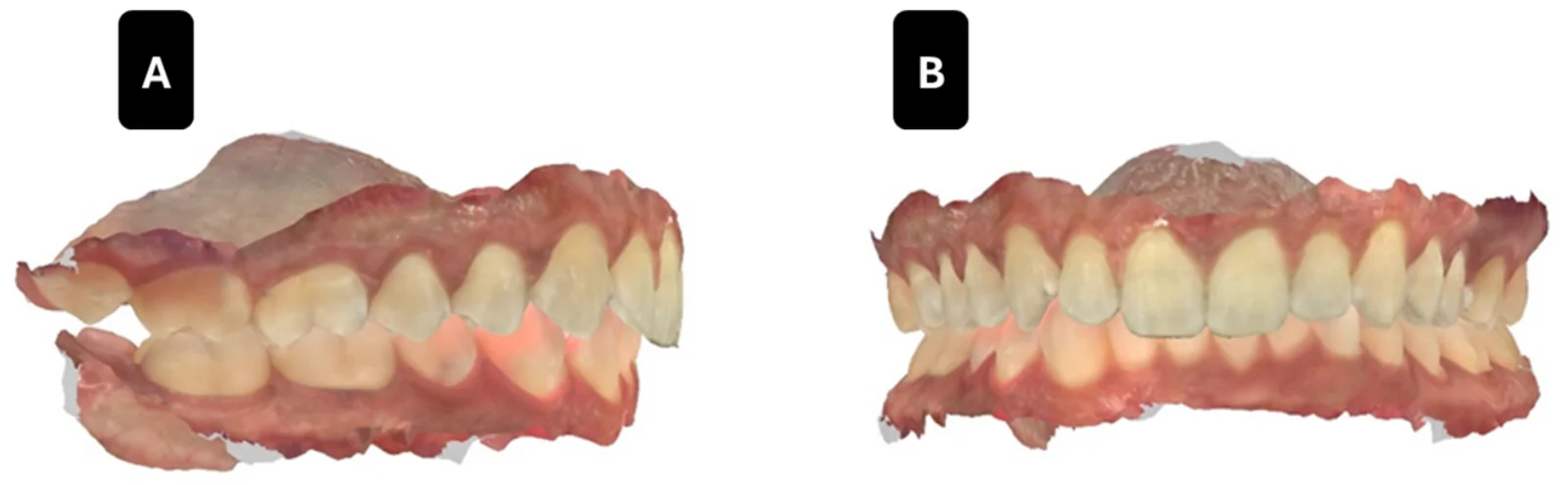
Intraoral digital scans displaying the occlusion from two perspectives: (**A**) lateral view bite, (**B**) frontal view.

Intraoral scanners (IOS), including systems like the Medit i700 and 3Shape TRIOS, are now commonly used in clinical dentistry for generating accurate, three-dimensional representations of the dental arches. These scanners are particularly effective in capturing fine surface detail and static occlusal contacts in maximum intercuspation (Figure 7). However, their ability to assess functional occlusion remains limited. On their own, IOS devices cannot register the sequence, timing, or intensity of occlusal contacts, factors that are critical for diagnosing occlusal imbalances and monitoring dynamic mandibular function [53].

Although IOSs can visualize areas of occlusal contact and facilitate virtual articulation, several studies have shown that they may lack consistency when it comes to identifying lighter or premature contacts. A study compared the Medit i600 with the T-Scan III and found that while the scanner offered a reliable visual overview of contact areas, it could not measure force dynamics or contact timing [66]. Similarly, another study reported that the CEREC Omnicam detected stronger contacts effectively, but its sensitivity decreased for lighter ones [67]. Moreover, another study confirmed that T-Scan provided more accurate and reproducible data on occlusal contact areas and timing, reinforcing its value when used alongside intraoral scanners in a complementary workflow [61].

IOS technologies also play a central role in recording the spatial relationship between the maxilla and mandible. By aligning previously captured upper and lower arch scans, these systems create a virtual articulation of the occlusion that can be used for diagnostic purposes and treatment planning [61,68]. They are especially useful when assessing static occlusion, although the quality of this digital articulation is influenced by multiple clinical and technical factors.

Several factors can influence the clinical performance of intraoral scanners, including operator experience, scanning strategy, patient cooperation, and the characteristics of the scanner itself. In addition to affecting scanning accuracy, these variables may also influence scanning efficiency and patient experience. An in vivo comparison of different intraoral scanners demonstrated differences in accuracy, scanning time, and patient comfort among the evaluated systems, highlighting the multifactorial nature of intraoral scanning performance in clinical practice [69].

A systematic review reported that IOS systems can produce static articulations comparable to conventional methods under ideal conditions, particularly in fully dentate arches with stable occlusal relationships [68]. However, their performance tends to decline in more complex cases, such as those involving multiple missing teeth or long-span prostheses [70]. Factors such as the articulation technique, the number and position of occlusal records, and the length of the scanned arches have all been shown to influence the reliability of digital occlusion [71,72].

In an in vitro study, the number of teeth included in the bilateral occlusal record had a measurable effect on articulation accuracy. Records that incorporated at least four teeth were significantly more accurate than those involving only two or three [70]. This finding has clinical implications for full-arch digital workflows, particularly in implant-supported prostheses or cases involving occlusal vertical dimension alterations.

From a technical perspective, the accuracy of intraoral scanners varies considerably depending on the scanner system, scanning protocol, arch length, and clinical conditions. Clinical studies evaluating complete-arch scans have reported trueness values ranging from 73 to 433 μm and precision values between 80 and 199 μm [61]. Notably, Kernen et al. reported trueness values of 433 ± 153 μm for the True Definition scanner, 147 ± 80 μm for the TRIOS system, and 198 ± 198 μm for the Omnicam system during complete-arch clinical scanning, highlighting substantial differences among IOS devices [73]. Furthermore, complete-arch scans generally demonstrate lower accuracy than short-span or half-arch scans due to cumulative stitching errors and operator-dependent factors. While these values are generally acceptable for many prosthodontic applications, they may not satisfy the requirements of high-precision occlusal force analysis. Consequently, IOS-based occlusal evaluation should be considered an adjunct to, rather than a replacement for, dedicated digital occlusal analysis systems within a comprehensive digital workflow.

When integrated with computerized occlusal analysis systems such as T-Scan, IOSs form part of a hybrid approach that combines surface accuracy with dynamic functional data. This synergy allows clinicians to visualize occlusal patterns in real-time, evaluate contact force distribution, and make informed adjustments, particularly in cases involving bruxism, temporomandibular disorders, or complex prosthodontic rehabilitation.

### 4.5. Innovative Technologies for Digital Occlusal Analysis and Jaw Tracking

Recent advancements have led to the development of various devices aimed at improving the assessment of occlusal forces, jaw movement, and muscle activity. The increasing need for dental rehabilitations as well as the need for precision have driven the adoption of several innovative technologies for evaluating occlusion. These advancements extend beyond static contact mapping and now incorporate tools that assess jaw motion, muscle activity, chewing analysis and occlusal dynamics in real time. The following subsections outline key systems currently used in clinical and research settings, each offering unique insights into occlusal function.

#### 4.5.1. Surface Electromyography

Surface electromyography (sEMG) has gained clinical relevance in assessing muscle activity during functional movements [47,48]. One such system, Teethan, measures the electrical signals of the masseter and anterior temporalis muscles during clenching. It utilizes four external probes that transmit data wirelessly to a receiver, which then displays processed results through dedicated software. These results include a numerical occlusal index, which can be used to evaluate muscle balance.

A study was previously conducted to assess Teethan’s ability. The clinical study involving 30 fully dentate participants reported stable index values across repeated clenching trials, with and without cotton rolls, at five-minute intervals, suggesting good reliability for baseline muscular assessments [47]. While the findings are promising, it remains essential to explore the diagnostic significance across diverse patient groups, especially those undergoing orthodontic or prosthodontic treatment.

#### 4.5.2. Four-Dimensional Jaw Tracking

**Figure 8:**
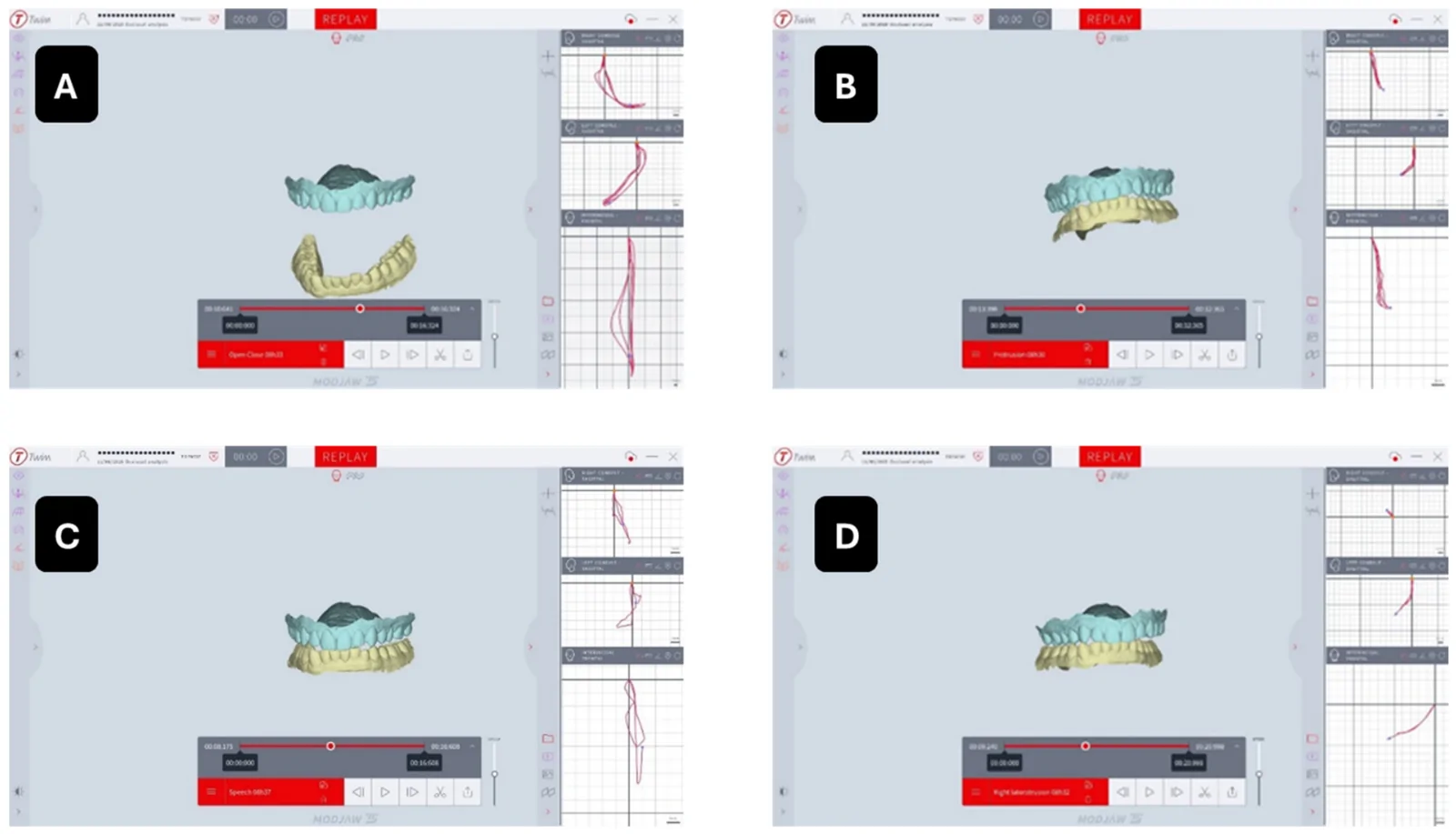
Modjaw device used for 4D tracking and analysis of mandibular function. (**A**) Mandibular opening and closing movements. (**B**) Protrusive movements, including lateral ones and pathways. (**C**,**D**) Right and left lateral excursion.

In contrast to sEMG, newer technologies encompassing four-dimensional jaw-tracking focus on dynamic mandibular movement. While jaw-tracking devices such as Cadiax, Modjaw, ARCUSdigma, and Zebris are often integrated into digital workflows, it is important to clarify that their primary role is to capture mandibular movement rather than directly assess occlusal contact. For example, Modjaw is a commonly used 4D jaw-tracking system that employs optical markers to record real-time jaw motion, including lateral excursions, protrusion, and chewing cycles (Figure 8). Its integration with CAD/CAM systems enables clinicians to visualize occlusion dynamically and refine restorative designs accordingly.

Recent in vitro investigations using typodont models demonstrated that Modjaw can achieve high trueness and precision, although a slight decline in accuracy was noted with increased vertical separation [50]. Additionally, early clinical reports have shown promise in its use for full-mouth rehabilitations and splint fabrication [74]. A study also investigated the precision and reliability of three jaw-tracking systems: Modjaw, KaVo ARCUSdigma, and SDiMatriX [75]. The study involved 25 participants and assessed mandibular movements. These movements included protrusion, retrusion, and lateral excursions. Modjaw demonstrated comparable trueness and precision to ARCUSdigma across all measured movements, indicating that it is a clinically reliable tool for capturing functional jaw dynamics. The study concluded that Modjaw is suitable for digital workflow integration in prosthodontic and orthodontic treatment planning [75].

Cadiax, which is the abbreviated term for Computer-Aided Diagnostics of the Articulator and Jaw Movements, is a digital successor to the mechanical pantograph. It utilizes electronic axiography to record mandibular motion in all three spatial planes, registering key parameters including sagittal condylar inclination (SCI), Bennett angle, hinge axis position, and excursive movements.

The reliability of the Cadiax 2 system for recording functional mandibular movements has been investigated in recent studies. The findings showed good repeatability for key condylar parameters, including the condylar slope and Bennett angle, across repeated recordings [76]. When compared with Modjaw, Cadiax produced slightly lower sagittal condylar inclination (SCI) values; however, the differences were considered clinically acceptable [77]. These results support the use of Cadiax as a reliable tool for mandibular movement analysis. Accurate recording of condylar movements is particularly important in full-mouth rehabilitation, where it can assist in more precise articulator programming and occlusal management. Comparative studies have confirmed that such discrepancies between systems are not uncommon, highlighting the importance of implementing standardized calibration protocols to minimize variability and improve consistency across digital jaw-tracking technologies [78,79]. Cadiax Compact 2, in particular, has been widely recognized for its clinical reliability, demonstrating excellent repeatability and low mean measurement error in condylographic recordings, making it a dependable tool for customized articulator programming and accurate functional diagnostics in prosthodontic treatment planning [80].

Overall, these systems provide valuable insights into functional pathways and condylar trajectories, aiding in motion simulation and prosthetic design. However, they do not directly assess occlusal contact timing, force distribution, or contact points, which are central to digital occlusal analysis. As such, they should be regarded as complementary tools rather than substitutes for systems like T-Scan or Accura, which are specifically designed to quantify occlusal forces and timing with clinical precision. Nevertheless, most of the reliability data for these jaw-tracking systems come from short-term, single-session recordings, and longitudinal studies evaluating their performance and clinical impact over time are still lacking.

A comparative overview of these systems is presented in Table 2, outlining their technology, recorded parameters, clinical applications, and practical considerations, thereby complementing the preceding discussion with a side-by-side summary of their core features.

## 5. Discussion

The accurate evaluation of occlusion plays a pivotal role in restorative and prosthodontic treatment, as diagnostic precision and proper adjustment directly influence long-term success. Conventional methods, including articulating paper, shimstock foil, sprays, and waxes, continue to be widely used because they are simple, inexpensive, and practical for daily clinical application. However, these visual indicators are inherently limited, particularly in complex restorative scenarios such as implant-supported prostheses, full-arch rehabilitations, and cases involving management of temporomandibular disorders (TMD), where quantification of occlusal force and timing becomes crucial.

A persistent misconception is that the size or intensity of articulating paper marks correlates directly with occlusal force. Different studies have evaluated different aspects of articulating paper performance, consistently demonstrating its limited ability to predict true occlusal load [31,32,81]. Carey et al. found no reliable relationship between articulating paper mark area and applied occlusal load [31], while Qadeer et al. reported that the largest articulating paper mark corresponded to the highest-force tooth in only 38.3% of quadrants [32]. Furthermore, Kerstein and Basson et al. summarized earlier evidence showing that a large articulating paper mark represented a forceful contact only 14% of the time and that clinicians correctly identified the highest-force contacts by visual interpretation of articulating paper markings in only 12.8–13.3% of assessments [35]. Collectively, these findings demonstrate that articulating paper is useful for identifying contact location but should not be relied upon to estimate occlusal force magnitude.

Conventional indicators are unable to capture dynamic parameters such as timing, sequence, and progression of contacts. These factors are critical for evaluating functional occlusion and stability, particularly in full-mouth rehabilitations and temporomandibular disorder (TMD) management. Although shimstock foil can confirm the presence or absence of contact, it provides no information regarding force magnitude or sequence [82]. Consequently, interpretation remains highly operator-dependent, compromising reproducibility [34]. Nevertheless, recent evidence suggests that the performance of articulating paper may be improved through standardized clinical protocols. Rovira-Lastra et al. reported good validity for articulating paper in detecting occlusal contact points, although the accuracy was influenced by paper thickness and clinical technique [83].

The clinical implications of these limitations are significant. Implant-supported prostheses are particularly vulnerable because of the absence of periodontal ligament feedback, increasing the risk of occlusal overloading. Similarly, high-strength ceramics such as zirconia and lithium disilicate, though esthetic and durable, are prone to chipping, marginal breakdown, and debonding under unbalanced occlusal forces [84,85]. A three-year randomized controlled trial on posterior implant-supported all-ceramic crowns highlighted the importance of regular occlusal monitoring and adjustment to maintain restoration integrity [86]. Another study comparing digital and conventional impression workflows reinforced the importance of accurate occlusal adjustment for optimizing restorative fit and long-term function [87].

Digital occlusal analysis systems have been developed to address these shortcomings by providing objective, quantifiable, and reproducible data on occlusal function. Systems such as T-Scan, OccluSense, and Modjaw enable clinicians to visualize occlusal force distribution, timing, and sequence in real time. Integrating T-Scan with intraoral scanners has been shown to enhance occlusal consistency and improve accuracy in full-arch rehabilitations and TMD management [53]. Its use in occlusal splint fabrication has also been associated with reduced chairside adjustment time [88]. OccluSense, with its more portable and clinician-oriented design, remains limited by the variability of pressure mapping and the absence of absolute force quantification [23]. Modjaw adds a dynamic dimension by capturing mandibular movements in four dimensions, offering valuable insight into functional occlusion despite its inability to directly measure occlusal force.

Nevertheless, digital systems are not without limitations. Most record relative rather than absolute force values, which can affect diagnostic precision. Sensor thickness may alter proprioceptive feedback or influence natural occlusal patterns, while repeated use can reduce sensitivity over time. Furthermore, intraoral visualization often still requires verification with articulating paper or foil to confirm contact locations [49]. Operator dependency also persists: accurate sensor positioning and appropriate data interpretation are essential, and variability tends to be higher among inexperienced users [23,61].

Furthermore, variations in recording protocols, sensor positioning, and data acquisition techniques may influence the accuracy and reproducibility of digital occlusal assessments [44,61,89]. Consequently, standardized workflows and adequate operator training are necessary to improve consistency and facilitate reliable interpretation of digital occlusal data [90]. These findings are consistent with the recent systematic review by Seth-Johansen and Gotfredsen [44], which reported generally acceptable reliability for digital occlusal analysis systems but less consistent validity for assessing contact intensity and exact interocclusal relationships. The authors concluded that digital occlusal technologies should currently be regarded as adjuncts rather than replacements for conventional occlusal assessment methods [44].

Cost, maintenance, and training requirements remain key barriers to wider clinical adoption. Supporting the reproducibility of digital assessment, Sigvardsson et al. [91] demonstrated high reliability for repeated digital occlusal contact measurements. However, the authors also reported weak agreement between digital contact area measurements and direct clinical examinations, suggesting that digital findings should be interpreted alongside clinical assessment rather than in isolation [91]. These limitations are further compounded by the predominance of laboratory-based studies, which may not fully reproduce clinical conditions. Although laboratory studies provide controlled conditions and facilitate standardization, they do not fully reproduce intraoral factors such as saliva, patient movement, periodontal ligament compliance, neuromuscular adaptation, and functional mandibular dynamics, which may influence occlusal contact detection and interpretation, highlighting the need for standardized protocols and well-designed clinical investigations [44,60,68]. This methodological variability may partly explain the inconsistent findings reported in systematic reviews regarding the agreement between digital and conventional methods for evaluating similar occlusal parameters [44,68].

Interpretation of the current evidence is further complicated by substantial methodological heterogeneity among studies, which makes direct comparison between conventional and digital occlusal analysis methods challenging. Conventional occlusal assessment encompasses a wide range of materials, including articulating papers of varying thicknesses, shimstock foil, occlusal sprays, silk strips, and silicone-based registration materials, each with distinct physical properties that record occlusal contacts differently [15]. Digital workflows are similarly heterogeneous, with some studies relying on intraoral scanner-based virtual occlusion, others evaluating scanned bite registrations, and others using computerized occlusal analysis systems or jaw-tracking technologies [15,23,72]. Studies that assess digital occlusion by scanning silicone or bite-registration materials introduce additional sources of error related to impression making, image acquisition, alignment procedures, and software-based interpretation of the occlusal record [71]. Operator-related factors, including scan path, scanner angulation, interocclusal record acquisition, and experience with digital workflows, further influence the accuracy and reproducibility of virtual occlusal assessments [61,70]. Comparison across studies is further limited by differences in study design, as many investigations have been conducted under laboratory conditions using typodonts or mounted casts [92,93] rather than under clinical conditions. Further validation is needed to confirm reproducibility during functional activities such as mastication, speech, and parafunction. Consequently, the reported accuracy of digital methods may be influenced not only by the underlying technology itself but also by the workflow used to generate and process the digital record [94].

While digital occlusal analysis systems are associated with several limitations, they provide additional information regarding occlusal timing, contact sequence, and relative force distribution that is not available from conventional indicators. These capabilities may be particularly valuable in complex clinical situations such as implant-supported restorations, full-mouth rehabilitations, and temporomandibular disorder management, where a comprehensive understanding of occlusal function is required. However, the extent to which these additional data translate into improved clinical outcomes remains incompletely established, and further well-designed clinical studies are needed.

Conventional and digital methods should therefore be regarded as complementary rather than interchangeable approaches. Conventional indicators remain valuable because they provide immediate intraoral visualization of contact location and are simple, inexpensive, and widely accessible. Digital systems, in contrast, provide additional quantitative information regarding force distribution and occlusal timing. While a combined approach may be clinically useful in selected cases, current evidence remains insufficient to conclude that combining conventional and digital methods consistently improves treatment outcomes compared with either method used independently. Future integration with digital articulators, jaw-tracking devices, and virtual patient workflows may further enhance occlusal assessment, although additional validation is required before widespread clinical implementation.

At present, neither conventional nor digital occlusal analysis methods can be considered a definitive gold standard for all clinical situations. Rather, the available evidence suggests that the selection of an occlusal assessment method should be guided by the clinical objective while recognizing the strengths and limitations inherent to each approach [23,44].

An important limitation of the current evidence base is that most studies have focused on fixed prosthodontic applications, including crowns, bridges, and implant-supported restorations. Comparatively little evidence is available regarding the use of digital occlusal analysis in removable prosthodontics, despite the importance of occlusal balance and functional stability in complete and partial denture rehabilitation. Further research is therefore needed to evaluate the role of digital occlusal assessment in removable prosthodontic treatment and determine whether findings from fixed prosthodontics can be generalized to these patient populations.

Furthermore, differences in scanning protocols, software algorithms, threshold definitions, and outcome reporting may contribute to variability in diagnostic performance. As a result, observed differences between conventional and digital methods may partly reflect methodological variation rather than true differences in clinical accuracy. Future directions point toward the incorporation of artificial intelligence (AI) and machine learning in digital occlusal analysis. These technologies can facilitate the automated identification of premature contacts, predict wear patterns, and optimize prosthetic designs by correlating occlusal data with anatomical and functional parameters. The concept of the virtual patient combining occlusal data with CBCT, facial scanning, and intraoral scans will further enhance interdisciplinary planning and personalized rehabilitation. Additionally, future research should focus on standardizing measurement protocols, improving sensor calibration, and validating reproducibility across different clinical conditions. Expanding applications beyond prosthodontics, such as to orthodontics, temporomandibular disorders, and musculoskeletal research, may also help establish occlusal analysis as a key component of comprehensive oral and systemic health evaluation.

## 6. Conclusions

Conventional methods for occlusal analysis remain indispensable in daily clinical practice because of their simplicity, affordability, and accessibility. However, their inherent subjectivity highlights the need for more objective and quantifiable approaches. Digital occlusal analysis systems provide valuable information on force distribution, occlusal timing, and mandibular dynamics, representing a significant advancement in diagnostic capability. At present, the most practical approach is the combined use of conventional indicators for intraoral verification and digital technologies for quantitative assessment. Future research should prioritize prospective longitudinal studies to establish the long-term reliability and clinical validity of digital occlusal analysis systems and determine whether changes in digitally recorded parameters, such as occlusal force, timing, contact distribution, and mandibular movement, translate into meaningful patient and prosthetic outcomes. Standardized protocols for device calibration, data acquisition, repeated measurements, and interpretation are also required to improve reproducibility and comparability across operators, devices, and clinical settings. Further research should evaluate the integration of occlusal data with intraoral scanning, CAD/CAM, virtual articulation, and dynamic jaw-tracking systems, particularly to determine whether incorporating these data into the virtual patient can improve treatment planning, prosthesis design, and clinical outcomes.

As digital occlusal systems become increasingly incorporated into restorative and prosthodontic workflows, greater emphasis should be placed on structured training, competency-based education, and standardized clinical protocols to ensure reliable data acquisition and interpretation. These considerations will be essential for translating technological advances into consistent clinical outcomes. With continued technological development and the integration of artificial intelligence, digital occlusal analysis is likely to become a routine component of clinical practice, contributing to improved treatment accuracy, enhanced patient outcomes, and greater long-term prosthetic success.

## Figures and Tables

**Table 1:** Summary of conventional occlusal analysis methods.

Material	Type/Brand	Thickness	Function/Application	Features	Limitations
Articulating Paper	Bausch BK 01, PROGRESS 100	100–200 μm	Assessment of occlusal contacts in static and dynamic occlusion	Progressive color transfer, two-sided, and comes as U-shaped as well as sectional	Subjective interpretation: poor correlation between mark size/intensity and occlusal force
Articulating Silk	Bausch Articulating Silk	80 μm	Assessment of occlusal contacts	High tear resistance	Subjective interpretation; does not provide force or timing information
Micro-Thin Articulating Paper	Arti-Check	40 μm	Detailed occlusal contact mapping in moist conditions	Double-sided liquid coating and can be used with restorations	Cannot quantify occlusal force, operator-dependent
Occlusion Test Films	Arti-Fol Shimstock Film	12 μm	Detailed contact assessment and interproximal analysis	Flexible, available with and without color coating; tear-resistant	Confirms contact only; does not identify force magnitude or timing
Ultra-Thin Film	Arti-Fol	8 μm	Detailed analysis with excursions	Different colors for ease of detection (excursions and protrusion)	Technique-sensitive; does not quantify occlusal force
Occlusion Spray	Arti-Spray	Thin spray	Detection of occlusal contacts and fit	Accurate and can be used with indirect prosthesis	Saliva- and application-sensitive; provides qualitative information only

**Table 2:** A comparative overview of widely used digital occlusal analysis and jaw-tracking systems.

System	Technology Type	Measures	Output	Clinical Applications	Considerations
T-Scan	Piezoelectric digital force sensor	Force, contact timing, sequence, and location	2D/3D visualization of force, sequence, and intensity	Prosthodontics, implantology, orthodontics, bruxism management	Cost, calibration needs, and operator training requirements
OccluSense	Wireless pressure-sensitive foil	Qualitative static and dynamic occlusal contacts	Tablet-displayed contact maps with color-coded feedback	Routine occlusal adjustments in general dentistry	Basic analytic functions and lower spatial resolution
Accura	Static pressure-sensitive mapping	Static occlusal force and contact area	Graphical representation of contact area and force levels	Pre-restorative occlusal screening and documentation	Lack of dynamic timing data and limited functional scope
Modjaw	4D optical mandibular motion tracking	Mandibular movement and occlusal dynamics	Real-time dynamic occlusion and functional motion capture	Comprehensive functional diagnostics in full-mouth rehabilitation	High-end system with ongoing clinical validation needed
Teethan	Surface electromyography (sEMG)	Muscle activity in masseter and temporalis	EMG indexes and muscle activity graphs	Pre-treatment neuromuscular assessment in TMD cases	Indirect occlusal assessment, not based on contact surfaces
Dental Prescale	Pressure-sensitive film	Occlusal contact area and bite force	Printed bite force maps with quantifiable pressure zones	Implant load monitoring and parafunctional habit analysis	Static-only recordings without live interaction capabilities
Zebris	Infrared-based mandibular tracking	Mandibular motion and condylar pathways	Functional simulations within CAD/CAM systems	Digital articulation and motion-guided prosthetic planning	Specialized integration needed with limited longitudinal data
ARCUSdigma	Ultrasonic and infrared motion tracking	Mandibular movement and hinge axis registration	Articulator integration with hinge axis and condylar tracing	Jaw tracking for advanced diagnostic and restorative workflows	Primarily research-supported; additional clinical data desirable

## Data Availability

Data are available upon request.
